# Supplementary material for: Practical Utility of a Clinical Pathway for Older Patients with Aspiration Pneumonia: A Single-Center Retrospective Observational Study
Source: J Clin Med. 2023 Dec 30;13(1):230. doi: 10.3390/jcm13010230 (PMC10779523; doi:10.3390/jcm13010230)
Supplement: Supplementary file 1 [file jcm-13-00230-s001.zip › Tables S1-S3.pdf]

Table S1 A-DROP scoring system for evaluating disease severity.

Parameter	Scoring
A: Age	Male ≥ 70 years, Female ≥ 75 years → +1 point
D: Dehydration	Blood urea nitrogen ≥ 21 mg/dL or dehydration → +1 point
R: Respiration	SpO_2 ≤ 90% or PaO_2 ≤ 60 Torr → +1 point
O: Orientation	Disturbance of consciousness → +1 point
P: Pressure	Systolic blood pressure ≤ 90mmHg → +1point
**Evaluation, points**	
0	Mild
1-2	Moderate
3	Severe
4-5	Most severe

Abbreviations: SpO_2, percutaneous oxygen saturation; PaO_2, arterial O_2 pressure.

Table S2 Sputum culture results on admission

Isolated pathogen	Non-CPW n=429	CPW n=167
Staphylococcus aureus	125 (29.1)	46 (27.5)
Klebsiella pneumoniae	59 (13.8)	15 (9.0)
Methicillin-resistant *Staphylococcus aureus*	54 (12.6)	15 (9.0)
Escherichia coli	51 (11.9)	15 (9.0)
Pseudomonas aeruginosa	38 (8.9)	14 (8.4)
Streptococcus pneumoniae	25 (5.8)	17 (10.2)
ESBLs *producing gram-negative bacilli*	21 (4.9)	3 (1.8)
Enterobacter cloacae	20 (4.7)	9 (5.4)
Moraxella catarrhalis	20 (4.7)	8 (4.8)
Haemophilus influenzae	13 (3.0)	9 (5.4)
Klebsiella oxytoca	13 (3.0)	7 (4.2)
Acinetobacter baumannii	9 (2.1)	2 (1.2)
Proteus millabillis	5 (1.2)	1 (0.6)
Serratia marcescense	5 (1.2)	0 (0)
Enterococcus faecalis	3 (0.7)	1 (0.6)
Stenotrophomonas maltophilia	2 (0.5)	2 (1.2)
Enterococcus faceum	1 (0.2)	0 (0.0)
PDR pathogen	106 (24.7)	30 (18.0)
Without sampling on admission	44 (10.3)	22 (13.2)

Abbreviations: ESBL, extended-spectrum β-Lactamase; PDR, potentially drug-resistant.

Table S3 Multivariable logistic regression analysis for mortality event

Variable	OR	95% CI	*P*
Age ≥ 87 years (vs. < 87 years)	0.92	0.58-1.47	0.72
Albumin ≤ 3.0 g/dL (vs. > 3.0 g/dL)	2.43	1.45-4.05	<0.001
Body mass index ≤ 17.8 kg/m^2 (vs. > 17.8 kg/m^2)	4.47	2.59-7.74	<0.001
A-DROP 3-5 points (vs. 0-2 points)	2.13	1.32-3.43	0.002
Resident in own home (vs. other location)	1.02	0.63-1.64	0.94
PDR detection (vs. without)	1.02	0.59-1.74	0.95
Clinical pathway use (vs. without)	0.75	0.43-1.30	0.30

Abbreviations: OR, odds ratio; CI, confidence interval; PDR, potentially drug-resistant.
