# Supplementary figures and images for: Practical Utility of a Clinical Pathway for Older Patients with Aspiration Pneumonia: A Single-Center Retrospective Observational Study
Source: J Clin Med. 2023 Dec 30;13(1):230. doi: 10.3390/jcm13010230 (PMC10779523; doi:10.3390/jcm13010230)

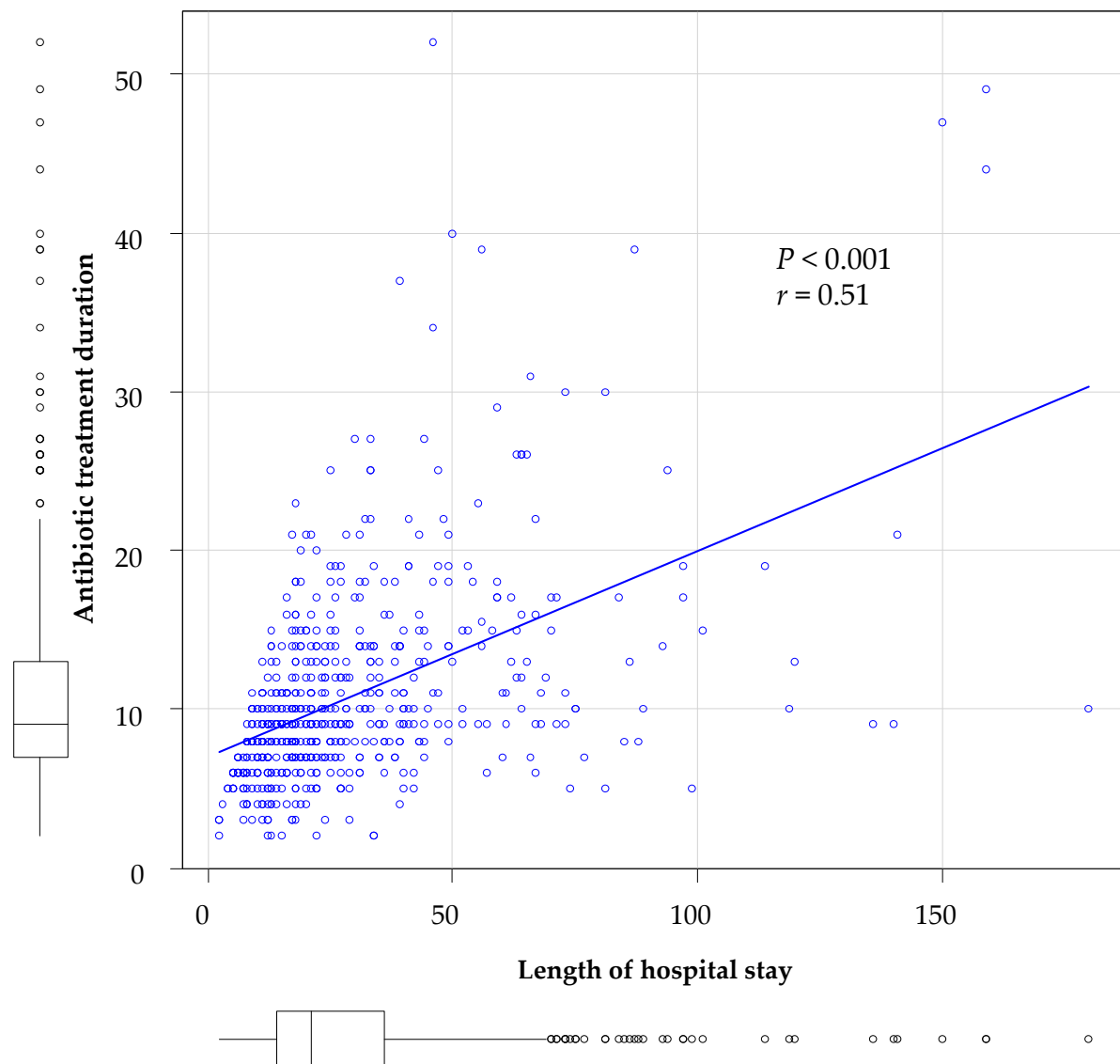

**Figure S1** Correlation between length of hospital stay and antibiotic treatment duration

Supplement: Supplementary file 1 [file jcm-13-00230-s001.zip › Figure S1.pdf]
